# Radial n-i-p structure SiNW-based microcrystalline silicon thin-film solar cells on flexible stainless steel

**DOI:** 10.1186/1556-276X-7-621

**Published:** 2012-11-12

**Authors:** Xiaobing Xie, Xiangbo Zeng, Ping Yang, Hao Li, Jingyan Li, Xiaodong Zhang, Qiming Wang

**Affiliations:** 1 State Key Laboratory on Integrated Optoelectronics, Institute of Semiconductors, Chinese Academy of Sciences, Beijing, 100083, China

**Keywords:** Silicon nanowires, Microcrystalline, Solar cells

## Abstract

Radial n-i-p structure silicon nanowire (SiNW)-based microcrystalline silicon thin-film solar cells on stainless steel foil was fabricated by plasma-enhanced chemical vapor deposition. The SiNW solar cell displays very low optical reflectance (approximately 15% on average) over a broad range of wavelengths (400 to 1,100 nm). The initial SiNW-based microcrystalline (μc-Si:H) thin-film solar cell has an open-circuit voltage of 0.37 V, short-circuit current density of 13.36 mA/cm^2^, fill factor of 0.3, and conversion efficiency of 1.48%. After acid treatment, the performance of the modified SiNW-based μc-Si:H thin-film solar cell has been improved remarkably with an open-circuit voltage of 0.48 V, short-circuit current density of 13.42 mA/cm^2^, fill factor of 0.35, and conversion efficiency of 2.25%. The external quantum efficiency measurements show that the external quantum efficiency response of SiNW solar cells is improved greatly in the wavelength range of 630 to 900 nm compared to the corresponding planar film solar cells.

## Background

Solar power as the richest clean energy is the most favorable substitution for biochemical energy resource, which would be exhausted in decades. Finding an effective and low-cost approach to harness solar energy is a key step to resolve the energy crisis [1]. Silicon thin-film solar cell technologies are industrially proven, environmentally friendly, and without fundamental limitation in material supply. However, the conflict between light absorption and photogenerated charge extraction makes planar silicon thin-film solar cells with comparatively low efficiencies [2]. Building radial junction thin-film solar cells on top of silicon nanowires (SiNWs) would enable a decoupling of the requirements for light absorption and carrier extraction into orthogonal spatial directions [3,4]. Also, the natural-light-trapping structure of SiNWs allows enhanced optical anti-reflection and absorption in a wide spectrum range [5-11]. Thus, the SiNW-based thin-film solar cells would be a potential candidate for low-cost and high-efficiency solar cells. The numerical simulation by Pei et al. shows an 11.6% conversion efficiency of SiNW-based thin-film solar cells [12]. The group of Yu has achieved some excellent experiment results in SiNW-based thin-film solar cells [13-15]. However, they almost focused on SiNW-based amorphous (a-Si:H) thin-film solar cells on glass substrates. In this work, we present SiNW-based microcrystalline (μc-Si:H) thin-film solar cells on flexible stainless steel substrates. The microcrystalline silicon film is an ideal light-absorbing material for solar cells due to its better stability under light soaking and stronger long-wavelength absorption compared to a-Si:H film [16]. Also, the μc-Si:H with a lower bandgap is more suitable for bandgap match between n-type crystalline SiNWs and i-type absorption layer [14]. In addition, flexible substrates make cells with a wider range of application.

## Methods

### n-SiNW synthesis

The indium nanoparticles as metal catalysts were fabricated by hydrogen plasma treatment on the indium tin oxide (ITO)-coated substrates [17], and the n-type SiNW growth is performed under plasma-enhanced chemical vapor deposition (PECVD) using SiH_4_ + PH_3_ as the precursor gas and H_2_ as the carrier gas [18]. The flexible stainless steel substrates were cleaned sequentially in baths of acetone, ethanol, and deionized water with ultrasonic agitation for 10 min. Then, they were coated with about 10-nm thick ITO by radio frequency magnetron sputtering. Subsequently, the ITO-coated substrates were loaded into the vacuum chamber of the PECVD. To fabricate indium nanoparticles as metal catalysts, the substrates were heated from room temperature to 400°C in 1.5 h in the vacuum chamber with a pressure of 1 to 2 × 10^−5^ Torr. Then, hydrogen plasma was irradiated on the substrates for 10 min with radio frequency (13.56 MHz) power density of 500 mW/cm^2^, a gas pressure of 1.5 Torr, and H_2_ gas of 60 sccm. The indium nanoparticle catalysts were attained after hydrogen plasma treatment. The pressure of the chamber was turned back to 1 to 2 × 10^−5^ Torr as soon as possible after hydrogen plasma treatment, and then, SiH_4_ + PH_3_ gas was introduced into the chamber as the Si source and doping source to synthesize the n-type SiNWs. Details of the synthesis conditions for the SiNWs are summarized in Table 1.

**Table 1:** Synthesis conditions for the n-type silicon nanowires

**Condition**	**Value**
SiH_4_ + PH_3_ flow rate	6 sccm + 6 sccm
H_2_ flow rate	60 sccm
Pressure	1.5 Torr
Substrate temperature	400°C
Radio frequency (13.56 MHz) power density	500 mW/cm^2^

SiH_4_ gas is 100% pure, and PH_3_ gas is 99 % H_2_ diluted.

### Acid treatment on n-type SiNWs

The as-synthesized n-type SiNW sample was taken out the PECVD chamber and dipped into 1% volume fraction hydrochloric acid for 10 min at room temperature. After the acid treatment, the sample was rinsed in deionized water for 10 min. Then, the air-dried sample was loaded into the PECVD chamber again for intrinsic and p-type layer coating.

### SiNW-based μc-Si thin-film solar cell formation

**Figure 1:**
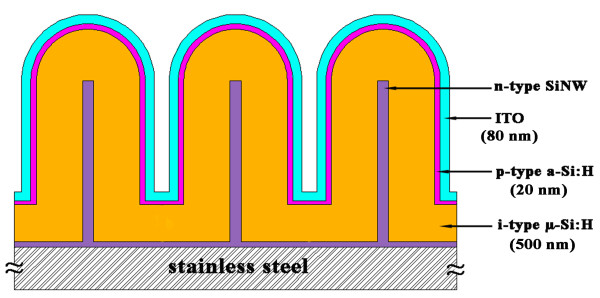
**The schematic structure of SiNW-based μc-Si:H thin-film solar cells.** n-type SiNWs coaxial covered by i-type μc-Si:H and p-type a-Si:H to form radial n-i-p structure.

The n-type SiNW samples with and without acid treatment were both loaded into the PECVD chamber. The base vacuum pressure for i-type μc-Si:H was 1 to 2 × 10^−6^ Torr, and the SiH_4_ + H_2_ (6 sccm + 60 sccm) gas was decomposed by a very high frequency (60 MHz) source with power density of 500 mW/cm^2^. The SiH_4_ + B_2_H_6_ gas is for p-type a-Si:H layer and decomposed by a radio frequency (13.56 MHz) source with power density of 1,000 mW/ cm^2^. After the p-type layer deposition, the samples were transferred to a radio frequency magnetron sputtering chamber for deposition of top ITO layer as front electrode. The sketch map of the SiNW-based μc-Si:H solar cell is shown in Figure 1.

## Results and discussion

### Morphology of n-type SiNWs and SiNW-based μc-Si:H thin-film solar cells

**Figure 2:**
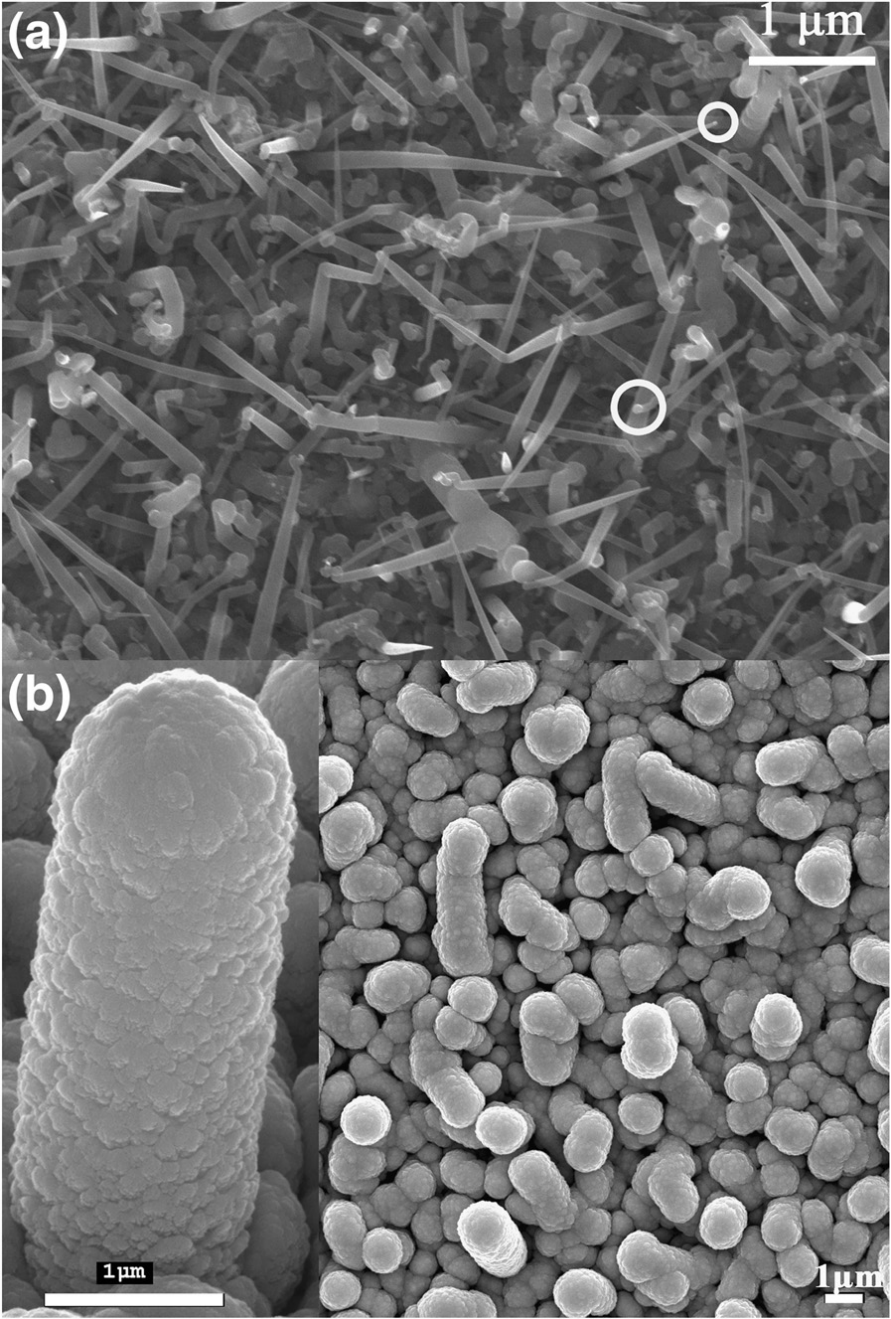
**SEM images of (a) the n-type SiNWs and (b) the SiNW-based μc-Si:H solar cells.** The bottom segment diameters of the SiNWs range from 50 to 200 nm, and their lengths extend about 3 μm. The white circles mark out the indium nanoparticles on top of the SiNWs. The final p-type a-Si:H/i-type μc-Si:H/n-type SiNW structure diameter is about 1.5 μm and rather uniform along its length.

Figure 2a shows the scanning electron microscopy (SEM) image of as-grown n-type SiNWs. The SiNWs were synthesized for 20 min on the flexible stainless steel substrates. The bottom segment diameters of the SiNWs range from 50 to 200 nm, and their lengths extend about 3 μm. The white circles mark out the indium nanoparticles on top of the SiNWs. The growth mechanism of the SiNWs may be interpreted basically by means of the vapor–liquid-solid (VLS) mechanism. Figure 2b shows the as-obtained SiNW-based μc-Si solar cells. As we can see, the final p-type a-Si:H/i-type μc-Si:H/n-type SiNW structure diameter is about 1.5 μm and rather uniform along its length. Considering that the SiNW diameter is typically much smaller than the thickness of the i-type μc-Si:H covering layer, we can assume that a conformal and uniform coverage on top of the rough SiNW structures has been achieved.

### Representation of acid treatment

**Figure 3:**
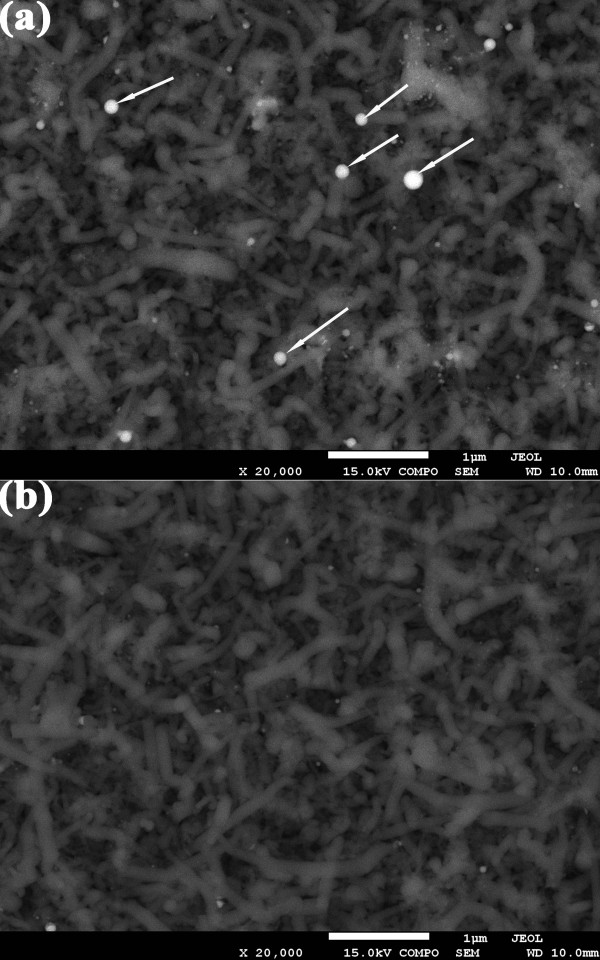
**Back-scattered electron SEM images of n-type SiNWs (a) before and (b) after acid treatment.** The white dots (marked by white arrows) denote the metal catalyst droplets for n-type SiNW VLS growth. After acid treatment, the white dots are almost absent.

Figure 3 presents the back-scattered electron SEM images of the n-type SiNWs before acid treatment (Figure 3a) and after acid treatment (Figure 3b). The white dots (marked by white arrows) denote the metal catalyst droplets for n-type SiNW VLS growth. As shown clearly, after acid treatment, the white dots are almost absent. It means that the metal catalyst droplets on the tip of n-type SiNW have been removed.

### The crystalline volume fraction of i-type μc-Si:H layer

**Figure 4:**
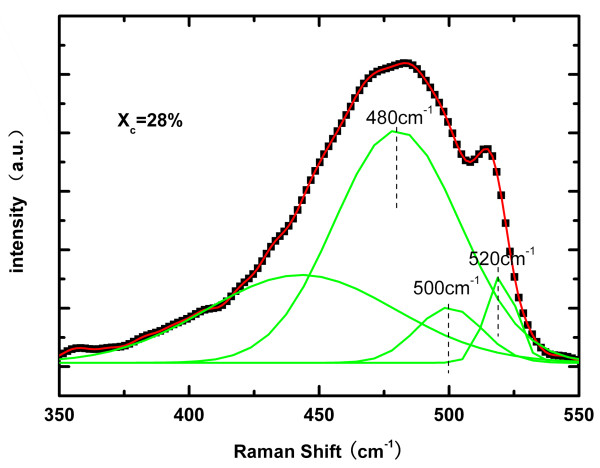
**Raman spectra of i-type μc-Si:H layer of the cells.** The Raman spectra of i-type μc-Si:H of the cells. The red line and line of black squares stand for the original measured data, and the green lines stand for the Gaussian fitting data. The Raman imaging microscope is equipped with a 514-nm wavelength line of an Ar laser.

Figure 4 shows the Raman spectra of i-type μc-Si:H of the cells. The Raman imaging microscope is equipped with a 514-nm wavelength line of an Ar laser. From the Raman spectra, the simple crystalline volume fraction (*X*_*c*_) of the sample could be calculated as follows [19,20]:

(1) $$X_{c}=\left(I_{520}+I_{500}\right)/\left(I_{520}+I_{500}+I_{480}\right)$$

where *I*_*i*_ is the area under the Gaussian centered at *i*, and *I*_*520*_*+ I*_*500*_*+ I*_*480*_ is the total integrated intensity. The values of *X*_*c*_ evaluated from the deconvoluted spectra is about 28%.

### Optical and electrical characterization

**Figure 5:**
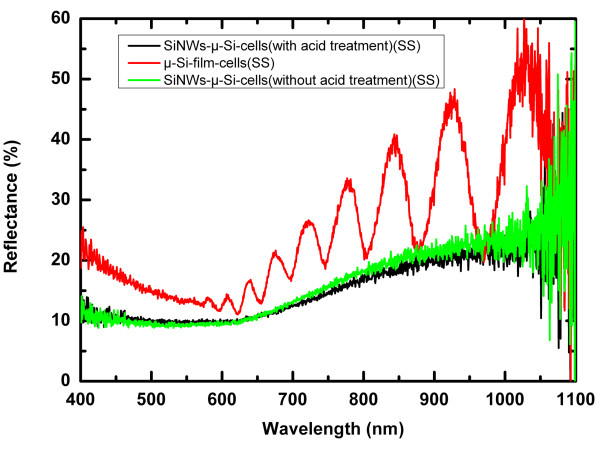
**Optical reflectance spectrum of the cells.** The optical reflectance spectrum of the cells over the full spectrum ranging from 400 to 1,100 nm. The black line and green line represent the spectra of the SiNW-based μc-Si:H thin-film solar cells with acid treatment and without acid treatment, respectively. The red line represents the reflectance of the corresponding planar μc-Si:H film solar cell.

**Figure 6:**
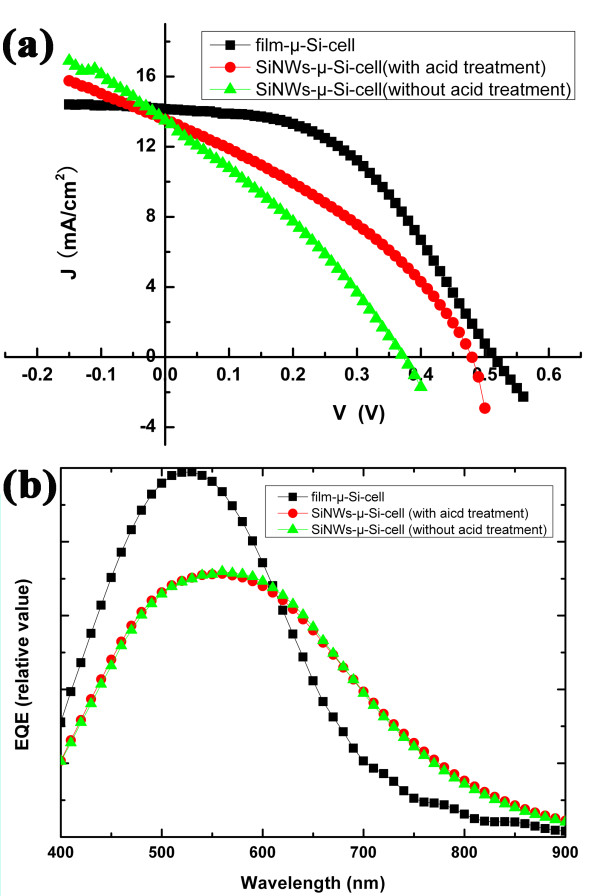
***I*-*V*and external quantum efficiency characteristics of the cells.** (**a**) Measured *I*-*V* characteristics of the SiNW-based solar cells (red dot line for cell with acid treatment and green triangle line for cell without acid treatment) and a corresponding planar silicon thin-film cell (black square line). (**b**) The external quantum efficiency of the SiNW-based solar cells and planar thin-film solar cell.

Figure 5 shows the optical reflectance spectrum of the cells over the full spectrum ranging from 400 to 1,100 nm. The black line and green line represent the spectra of the SiNW-based μc-Si:H thin-film solar cells with acid treatment and without acid treatment, respectively. As can be seen, the optical reflectance of SiNW-based μc-Si:H thin-film solar cells is below 20% in the full spectrum and only about 10% in the range of 400- to 650-nm wavelengths. The average reflectance is approximately 15%, which shows a 40% reduction compared to approximately 25% average reflectance of the corresponding planar μc-Si:H film solar cell (red line). It indicates that the low optical reflectance of the SiNW-based μc-Si:H thin-film solar cells is due to the light-trapping structure of SiNWs. Figure 6a shows the typical light current–voltage (*IV*) curves of the cells measured under a solar simulator with air mass (AM) 1.5 (100 mA/cm^2^) illumination in the 0.07-cm^2^ effective areas. The short-circuit current density (*J*_SC_), open-circuit voltage (*V*_OC_), fill factor (FF), and conversion efficiency (*η*) derived from the *IV* curve are listed in Table 2. The initial SiNW-based μc-Si:H thin-film solar cell has values of *V*_OC_ = 0.37 V, *J*_SC_ = 13.36 mA/cm^2^, FF = 0.3, and *η* = 1.48%. After acid treatment, the metal catalyst droplets have been removed from the top of the n-type SiNWs. The performance of the modified SiNW-based μc-Si:H thin-film solar cell has been improved remarkably with the *V*_OC_ = 0.48 V, *J*_SC_ = 13.42 mA/cm^2^, FF = 0.35, and *η* = 2.25%. It is worth mentioning that the *V*_OC_ improved by approximately 30% (from 0.37 to 0.48 V), and the *J*_SC_ improved only by 0.4% (from 13.36 to 13.42 mA/cm^2^). Considering the influence of the metal catalyst droplets in the SiNW-based μc-Si:H thin-film solar cells, we think that the metal catalyst droplets increase the interface of the n-type SiNW and i-type μc-Si:H layer. Thus, the interface recombination would increase when the metal catalyst droplets exist. The *V*_OC_ is most sensitive for interface recombination. So, it is most favorable for *V*_OC_ improvement when the metal catalyst droplets were removed. In addition, refer to Figure 5, the metal catalyst droplets almost do not affect the optical reflectance of the cells. That could explain why the modified SiNW-based μc-Si:H thin-film solar cell has little improvement in *J*_SC_. The current–voltage curve of the corresponding planar μc-Si:H film solar cell is also shown in Figure 6a (black solid square line) for contrast. As can be seen, the performance of the SiNW-based cells is not as good as that of the corresponding planar film cell, especially in terms of *V*_OC_ and FF. The unsatisfactory performance of the SiNW-based cell could be attributed to more interface recombination which brings higher leakage current and lower shunt resistance [21]. Thus, the interface passivation is the most important for SiNW-based thin-film solar cells.

**Table 2:** **Parameters of the cells for light-soaking *I*-*V *characters**

**Parameters**	**Cells**		
	**SiNWs cell (without acid treatment)**	**SiNWs cell (acid treatment)**	**Planar film cell**
*V*_oc_ (V)	0.37	0.48	0.51
*J*_sc_ (mA/cm^2^)	13.36	13.42	14
FF	0.3	0.35	0.46
*η* (%)	1.48	2.25	3.28

Measured under solar simulator with AM 1.5 (100 mA/cm^2^) illumination in the 0.07 cm^2^ effective area.

Figure 6b shows the external quantum efficiency (EQE) of the SiNW-based μc-Si:H solar cells (red dot line for cells with acid treatment and green triangle line for cells without acid treatment) and planar μc-Si:H film solar cell (black square line). From the picture, the EQE of the SiNW-based cells has a definite improvement in the wavelength range of 630 to 900 nm that is due to the light-trapping effect of SiNWs. However, in the short wavelengths (from 400 to 630 nm), the SiNW-based cells show lower EQE than the corresponding planar film cell, which is inconsistent with the results of the optical reflectance spectrum shown in Figure 5. It may be explained as follows: first, the anti-reflection effect of SiNW-based cells in short wavelengths is not as good as in long wavelengths (refer to Figure 5); second, the interface of the n-type SiNWs and i-type μc-Si:H layer influences the collection of photo-induced electrons, especially the photo-induced electrons in the short-wavelength range.

## Conclusions

We have produced a radial n-i-p structure SiNW-based μc-Si:H thin-film solar cell on stainless steel foil by plasma-enhanced chemical vapor deposition. The SiNW solar cell displays a very low optical reflectance over a broad range of wavelengths (400 to 1,100 nm) due to its natural anti-reflective structure. The modified open-circuit voltage, short-circuit current density, and conversion efficiency under AM 1.5 illumination were 0.48 V, 13.42 mA/cm^2^, and 2.25%, respectively. The EQE measurements show that the EQE response of SiNW solar cells is improved greatly in the wavelength range of 630 to 900 nm compared to the corresponding planar film solar cells. Further, we will focus on improving the SiNW solar cells by minimizing shunts, reducing contact resistance, and improving the open-circuit voltage.

## Abbreviations

EQE: external quantum efficiency; FF: fill factor; ITO: indium tin oxide; *J*_sc_: short-circuit current density; PECVD: plasma-enhanced chemical vapor deposition; SEM: scanning electron microscope; SiNW: silicon nanowire; VLS: vapor–liquid–solid; *V*_oc_: open-circuit voltage; μc-Si:H: Hydrogenated microcrystalline silicon.

## Competing interests

The authors declare that they have no competing interests.

## Authors’ contributions

XBZ and QW conceived of the study, participated in its design and coordination, and revised the manuscript. XX participated in its design and coordination, drafted the manuscript, and carried out the experiments on fabrication of SiNWs. PY carried out the measurement and analysis of optical and electoral characterization. HL carried out the measurement and analysis of the Raman spectrum. JL and XDZ carried out the PECVD experiments for silicon thin-film deposition. All authors read and approved the final manuscript.
